# Impact of Intensive Glucose Control in Patients with Diabetes Mellitus Undergoing Percutaneous Coronary Intervention: 3-Year Clinical Outcomes

**DOI:** 10.3390/jcm9082464

**Published:** 2020-08-01

**Authors:** Jiesuck Park, Jung-Kyu Han, Mineok Chang, You-Jeong Ki, Jeehoon Kang, Han-Mo Yang, Hyun-Jai Cho, Kyung Woo Park, Hyun-Jae Kang, Bon-Kwon Koo, Hyo-Soo Kim

**Affiliations:** Cardiovascular Center, Seoul National University Hospital, Seoul 03080, Korea; cardio.jspark@gmail.com (J.P.); hpcrates@gmail.com (J.-K.H.); oklizard81@gmail.com (M.C.); drkiyou@gmail.com (Y.-J.K.); medikang@gmail.com (J.K.); hanname@hanmail.net (H.-M.Y.); hyunjaicho@snu.ac.kr (H.-J.C.); kwparkmd@snu.ac.kr (K.W.P.); nowkang@snu.ac.kr (H.-J.K.); bkkoo@snu.ac.kr (B.-K.K.)

**Keywords:** diabetes mellitus, percutaneous coronary intervention, HbA1c, hyperglycemia

## Abstract

We investigated whether intensive glucose control after percutaneous coronary intervention (PCI) improves clinical outcomes in diabetic patients. From the Grand-DES registry, we analyzed 2576 diabetic patients (median age 66 years, male 65.6%) who underwent PCI and had at least 2 records of HbA1c during the follow-up. Patients were categorized according to the mean HbA1c (≥7% or <7%). Primary outcome was major adverse cardiovascular event (MACE), a composite of cardiac death, non-fatal myocardial infarction, and any revascularization. During a median follow-up of 33.6 months, MACE occurred in 335 (13.0%) patients. Intensive glucose control with follow-up mean HbA1c < 7.0% (42.2%; *n* = 1087) was not associated with lower risk of MACE, compared to control with mean HbA1c ≥ 7.0% (adjusted hazard ratio [aHR] [95% confidence interval] 1.06 [0.82–1.37], *p* = 0.672). In subgroup analysis, patients with sustained HbA1c of <7.0% throughout the follow-up were not associated with a lower risk of MACE compared to those with sustained HbA1c of ≥7.0% (aHR 1.15 [0.71–1.89], *p* = 0.566). More intensive glucose control with mean HbA1c ≤ 6.5% was not associated with lower risk of MACE, compared to loose control with a mean HbA1c ≥ 8.0% (aHR 1.15 [0.71–1.86], *p* = 0.583). Intensive glucose control after PCI was not associated with better clinical outcomes in diabetic patients undergoing PCI than lenient control.

## 1. Introduction

Cardiovascular disease is one of the fatal complications among patients with diabetes mellitus (DM) [1]. In addition, around a quarter of coronary revascularization procedures are performed in patients with DM [1]. Patients with DM who underwent percutaneous coronary intervention (PCI) tend to be associated with worse clinical outcomes such as a higher risk of stent restenosis and major adverse cardiovascular events (MACE) compared with those without DM [2,3]. Diabetes mellitus is also associated with an increased risk of hospitalization for heart failure after PCI [4]. Therefore, optimal risk factor management in DM patients undergoing PCI is clinically relevant, and finding the ideal level of glycemic control, represented by the glycated hemoglobin (HbA1c) level, is of particular interest to clinicians.

Previous randomized trials raised questions regarding the benefit of intensive glucose control, which was generally defined as a target HbA1c < 6–7%, for the prevention of macrovascular events, especially in patients with long duration of diabetes or an advanced atherosclerotic burden [5,6,7]. In particular, it is not yet understood whether intensive glucose control improves the clinical outcomes particularly in patients with DM after PCI. Only a few registry-based studies reported the role of glucose control in these patients [8,9,10]. However, the sample size and follow-up duration of these studies were limited. Although some of these studies reported worse clinical outcomes associated with loose glucose control, which were mainly driven by repeat revascularization [9,10], these studies were based on single center experience and patients were categorized according to HbA1c measured at a single time point. Given the chronic nature of DM, the level of glucose throughout the long-term follow-up should be considered to find a legitimate answer for the role of glycemic control. Therefore, we collected data regarding participants’ HbA1c levels trimonthly after discharge in the prospective multicenter DES registries, and classified the patients according to the mean HbA1c derived from HbA1c values measured throughout the follow-up period. Then we sought to investigate the long-term impact of intensive glucose control on clinical outcomes in diabetic patients undergoing PCI.

## 2. Methods

### 2.1. Data Source and Study Population

**Figure 1:**
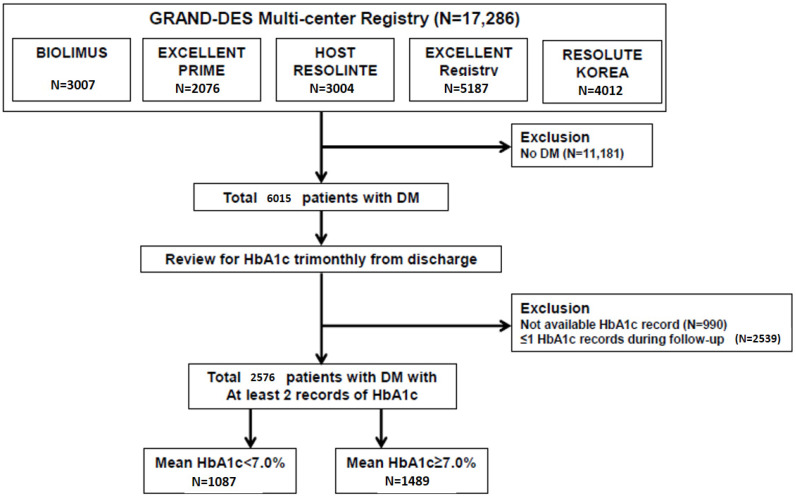
Study flow. From the Grand-DES registry, a patient-level pooled registry including five Korean multicenter prospective drug-eluting stent registries, a total of 17,286 patients with CAD, who had undergone PCI, were screened for inclusion in this study. After collecting the records of HbA1c level trimonthly after discharge, a total of 2576 patients with DM, who underwent PCI and had at least ≥2 records of HbA1c, were finally included. Patients were classified according to mean HbA1c cut-off value of 7%. CAD = coronary artery disease, DM = diabetes mellitus, PCI = percutaneous coronary intervention.

We selected the subjects from the Grand Drug-Eluting Stent (Grand-DES) registry (NCT03507205) which is a patient-level pooled registry comprising five prospective multicenter DES registries in the Republic of Korea that were moderated by Seoul National University Hospital: Harmonizing Optimal Strategy for Treatment of coronary artery disease using a BIOLIMUS A9-eluting stent (HOST-BIOLIMUS-3000-Korea) registry, Efficacy and Safety of Xience in Coronary artEry Disease aLL-comers After stENTing Using the PRIME Platform (EXCELLENT-PRIME) registry (NCT01605721), Harmonizing Optimal Strategy for Treatment of coronary artery disease using a RESOLute INTEgrity (HOST-RESOLINTE) registry, Efficacy of Xience/Promus versus Cypher in rEducing Late Loss after stenting (EXCELLENT) registry (NCT00698607), and Registry to Evaluate the Efficacy of Zotarolimus-Eluting Stent (RESOLUTE-Korea) (NCT00960908). A total of 17,286 patients were enrolled from 55 participating centers in the Republic of Korea. The institutional review boards at each participating center approved the study protocol including that of Seoul National University Hospital, and the study was conducted following the principles of the Declaration of Helsinki. Informed consent was obtained from all the participants before study enrolment. For the current study, we selected a total of 6105 patients with a history of DM who underwent PCI (Figure 1).

We assessed the post-PCI glycemic control status in each patient by reviewing their HbA1c records at intervals of three-month after discharge. Patients without available data regarding HbA1c or those with only single record of HbA1c after PCI were excluded. Finally, a total of 2576 DM patients, who had at least two records of HbA1c, were included in further analysis. The clinical data, such as demographic information, comorbidities, and records of coronary revascularization and medical treatment, were obtained from the database and analyzed.

### 2.2. Statistical Analysis

Using multiple records of HbA1c obtained after PCI, patients were categorized based on mean HbA1c cut-off value of 7% (mean HbA1c ≥ 7% or <7%). The primary outcome was MACE, a composite outcome consisting of cardiac death, non-fatal myocardial infarction (NFMI), and any revascularization. We also assessed each component of the primary outcome, all-cause death, and a composite of cardiac death and NFMI, stroke, and target lesion revascularization (TLR). The risk of clinical outcomes were compared between the groups using Kaplan–Meier survival analysis with log-rank test and multivariable-adjusted Cox hazard regression models based on the baseline characteristics of the study population: demographic information (age, sex, and body mass index), cardiovascular risk factors (previous history of hypertension, dyslipidemia, congestive heart failure or left ventricular dysfunction, chronic kidney disease, stroke, peripheral vascular disease, myocardial infarction [MI], previous PCI or coronary artery bypass graft, and current smoking), initial presentation as acute MI, coronary lesion and procedure characteristics (multiple coronary lesions, left main coronary artery disease, type B2/C lesion, calcification, stent generation, total number of stent, stent diameter, and total stent length), medication records (insulin, sulfonylurea, glinide, metformin, dipeptidyl peptidase-4 inhibitor, thiazolidinedione, a-glucosidase inhibitor, aspirin, clopidogrel, beta-blockers, angiotensin-converting enzyme inhibitors, angiotensin II receptor blockers, and statin), and laboratory results (baseline HbA1c, numbers of HbA1c records, total cholesterol, triglyceride, high-density lipoprotein, low-density lipoprotein, and creatinine clearance). Additionally, a sensitivity analysis was conducted after balancing the potential confounding factors between the groups by applying propensity score (PS) matching technique. In brief, the PS of being in each treatment group was calculated using an ordinary logistic regression based on all the baseline covariates included in the Cox regression analysis [11]. We considered the maximum absolute standardized difference of 0.1 (10%) as a negligible difference in baseline characteristics between the groups [12]. The event rates of the clinical outcomes were calculated before and after PS matching. Further subgroup analyses proceeded after the patients were categorized according to (1) HbA1c level sustained below or over 7.0% throughout the follow-up period to find the impact of stable glycemic control, and (2) mean HbA1c ≤ 6.5% or ≥8.0% to see the impact of more intensive glycemic control. All statistical analyses were performed using R version 3.4.3 (R Development Core Team, Vienna, Austria). All probability values were two-sided, and *p*-values < 0.05 were considered statistically significant.

## 3. Results

### 3.1. The Impact of Intensive Glucose Control with Mean HbA1c < 7.0% on Clinical Outcomes in Diabetic Patients after PCI: Whole Population

To accurately reflect the status of glycemic control after PCI, we retrospectively collected HbA1c data for each patient at intervals of three-month after discharge and calculated mean HbA1c. During the follow-up period, 6.0 ± 3.4 times of HbA1c records were obtained per each patient. Among a total of 2576 eligible patients, there were 1087 (42.2%) and 1489 (57.8%) patients with mean HbA1c < 7%, and ≥7%, respectively (Figure 1). Table 1 summarizes the baseline characteristics of the study population.

**Figure 2:**
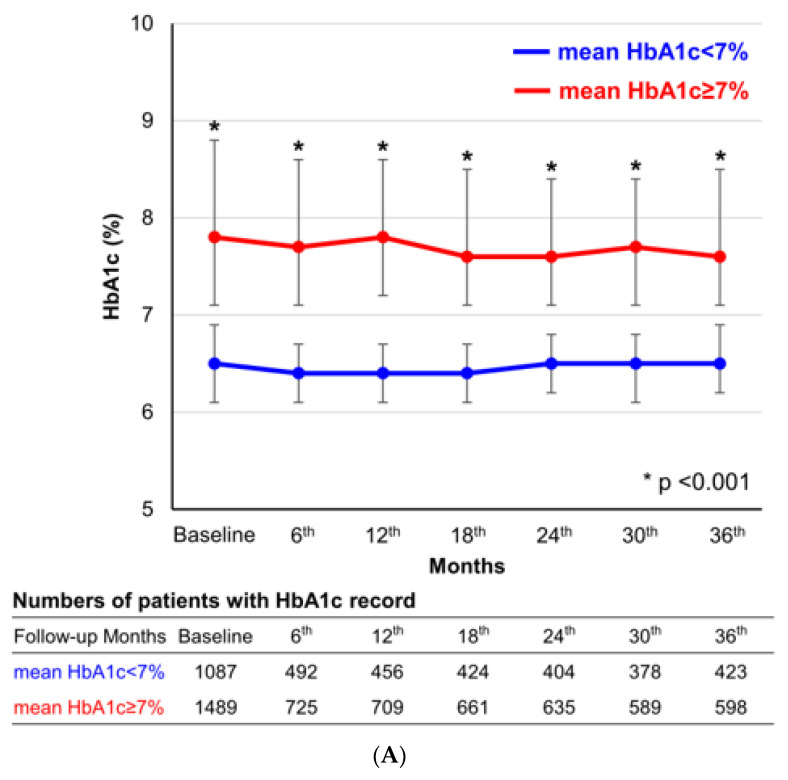

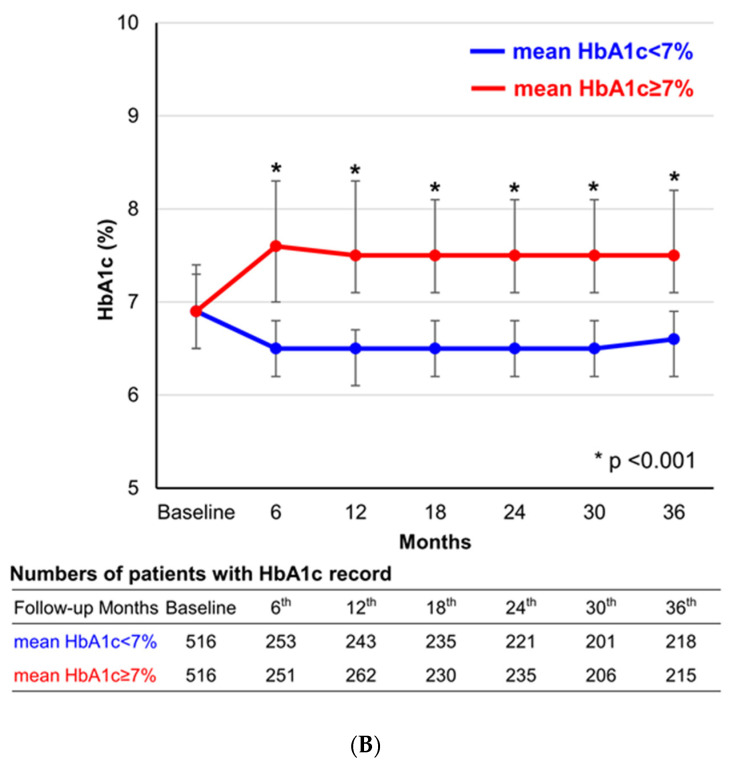
**Temporal changes in HbA1c in the overall and propensity score matched 516 paired populations.** The figures represent the temporal changes in HbA1c among the overall (**A**) and propensity score matched population (**B**). The blue and red lines represent the mean HbA1c < 7.0 group and mean HbA1c ≥ 7.0 group, respectively. The error bars represent the interquartile range of HbA1c level.

There were no significant differences in demographic features and comorbidities between the two groups. Mean HbA1 < 7% group had a higher prevalence of multivessel coronary disease, received 2nd generation DES implantation more frequently, and had a longer total stent length than mean HbA1c ≥ 7% group. Mean HbA1c < 7% group took sulfonylurea less frequently, compared with mean HbA1c ≥ 7% group. The baseline HbA1c was 6.5 ± 0.8%, and 8.1 ± 1.5% in mean HbA1c < 7%, and ≥7% group, respectively. Throughout the follow-up period, the HbA1c levels were significantly separated between the two groups (Figure 2A).

**Figure 3:**
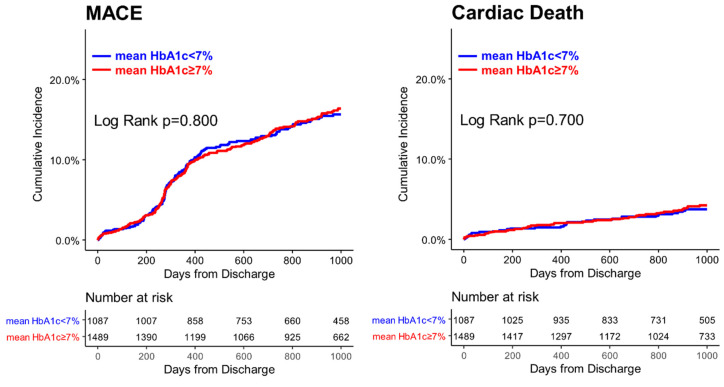
**Kaplan–Meier curve for clinical outcomes according to mean HbA1c cut-off of 7.0% in the overall populations.** Among the overall population, patients with mean HbA1c ≥ 7% was not associated with a better event-free survival in clinical outcomes compared with those with mean HbA1c < 7%. MACE = major adverse cardiovascular event.

During the median follow-up period of 33.6 months (interquartile range 21.6–37.2 months), MACE occurred in 13.0% (*n* = 335) of patients. In the overall population, intensive glucose control with mean HbA1c < 7.0% was not associated with better clinical outcomes, such as MACE (12.7% vs. 13.2% for mean HbA1c < 7.0% vs. ≥7% group, adjusted hazard ratio (aHR) 1.06, 95% confidence interval (CI) 0.82–1.37, *p* = 0.672), cardiac death (3.1% vs. 3.5% aHR 1.06, 95% CI 0.63–1.81, *p* = 0.820), non-fatal MI (0.6% vs. 1.5%, aHR 0.41, 95% CI 0.15–1.16, *p* = 0.093), and any revascularization (9.8% vs. 9.7%, aHR 1.05, 95% CI 0.78–1.42, *p* = 0.748), compared with glucose control with HbA1c ≥ 7% (Table 2, Figure 3). Other clinical outcomes, such as all-cause death, stroke, and TLR, showed the same trend.

### 3.2. The Impact of Intensive Glucose Control with Mean HbA1c < 7.0% on Clinical Outcomes in Diabetic Patients after PCI: Propensity-Matched 516 Pairs

After PS matching, the differences in baseline characteristics between the comparative groups were well balanced (Table 1, Figure S1). To discriminate the impact of post-PCI glycemic control, the baseline HbA1c level was also matched. During the follow-up period, the level of HbA1c was significantly separated between the two groups (Figure 2B).

The incidences of outcomes were also not different between the two groups after PS matching demonstrating that intensive glucose control after PCI was not associated with better clinical outcomes (Table 2), such as MACE (14.1% vs. 12.2% for mean HbA1c < 7.0% vs. ≥7% group, aHR 1.17, 95% CI 0.84–1.65, *p* = 0.351), cardiac death (4.1% vs. 2.3% aHR 1.76, 95% CI 0.87–3.36, *p* = 0.119), and non-fatal MI (0.6% vs. 1.2%, aHR 0.50, 95% CI 0.13–2.01, *p* = 0.330), compared with glucose control with HbA1c ≥ 7% after PCI.

### 3.3. Subgroup Analysis of MACE

**Figure 4:**
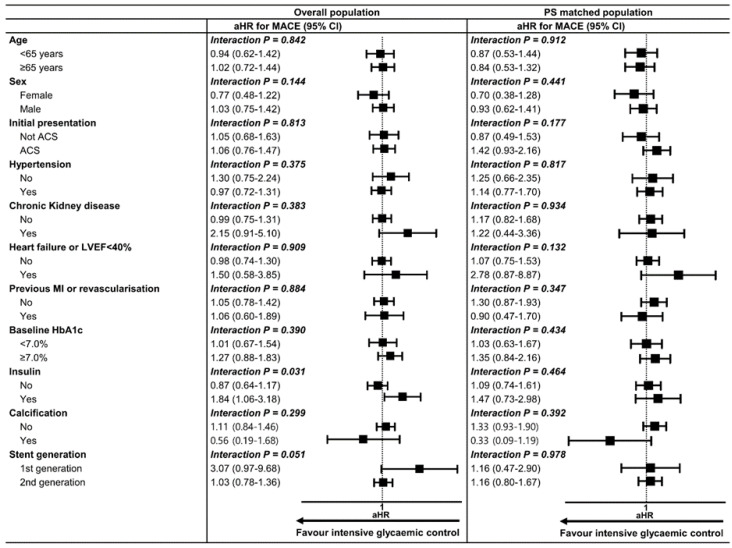
**Subgroup analysis of clinical outcomes according to mean HbA1c cut-off of 7.0%.** In subgroup analysis, mean HbA1c < 7.0% group showed no significant risk reduction for MACE compared with mean HbA1c ≥ 7.0% group across various subgroups except for those with insulin treatment. For subgroup with insulin treatment, HbA1c < 7.0% group was associated with a higher risk of MACE than HbA1c ≥ 7% group. ACS = acute coronary syndrome, CI = confidence interval, aHR = adjusted hazard ratio, LVEF = left ventricular ejection fraction, MACE = major adverse cardiovascular event, MI = myocardial infarction, PS = propensity score.

To determine whether the outcomes according to mean HbA1c throughout the follow-up period were consistent, we calculated aHR for MACE in various subgroups. The results showed no significant difference in the risk of MACE between mean HbA1c < 7.0% and ≥7% groups across most subgroups (Figure 4). Interestingly, among the patients receiving insulin, mean HbA1c < 7.0% group was associated with a higher risk for MACE with borderline significance compared with mean HbA1c ≥ 7% group. This finding could be related to the risk of hypoglycemic complications imposed by insulin usage.

### 3.4. The impact of ‘Stable’ Glucose Control on Clinical Outcome after PCI

To rule out the effects of fluctuation in glucose control, and to determine the impact of stably controlled glucose, the patients with sustained HbA1c of <7.0% or ≥7.0% throughout the follow-up period were selected. A total of 532 (20.7%), and 716 (27.8%) patients had sustained HbA1c of <7.0% and ≥7.0%, respectively (Table S1). Throughout the follow-up, the HbA1c levels were significantly separated between the two groups (Figure S2A). Compared with sustained HbA1c ≥ 7.0% group, sustained HbA1c < 7.0% group was not associated with better clinical outcomes, suggesting that even stably maintained intensive glucose control did not significantly improve the outcomes (Table 3, Figure S3A).

### 3.5. The Impact of ‘More Intensive’ Glucose Control on Clinical Outcome after PCI

Many guidelines would suggest more stringent HbA1c target ≤6.5% or less stringent one such as <8.0% or ≤9.0% on a personalized basis [13,14,15,16]. The Action in Diabetes and Vascular Disease: Preterax and Diamicron Modified Release Controlled Evaluation (ADVANCE) trial defined intensive glucose control as a target HbA1c value ≤ 6.5% in their study [5]. Therefore, we compared the clinical outcomes of mean HbA1c ≤ 6.5% group with that of mean HbA1c ≥ 8.0% group (Table S3). During the follow-up period, the HbA1c levels were significantly separated between the two groups (Figure S2B). The results revealed that more stringent glucose control with mean HbA1c < 6.5% was also not associated with better clinical outcomes, compared with less stringent control with mean HbA1c ≥ 8.0% (Table 4, Figure S3B).

## 4. Discussion

### 4.1. An HbA1c Goal to Reduce the Risk of Macrovascular Complications

Current guidelines generally recommend a target HbA1c ≤ 7.0% for the management of patients with stable coronary artery disease complicated by DM [13,14], or primary prevention in diabetic patients [15]. Less stringent HbA1c goals (such as 7–9%) were suggested for certain patients with the risk of hypoglycemia, cardiovascular complications, extensive comorbidity, advanced age, etc. However, evidence for an HbA1c goal to reduce the risk of macrovascular complications is not compelling, whilst a target HbA1c ≤ 7.0% has been shown to reduce microvascular events.

The UK Prospective Diabetes Study (UKPDS) showed that intensive glucose control (median HbA1c of 7.0%) in newly diagnosed diabetic patients decreased the risk of microvascular complications but not macrovascular disease during 10-year follow-up compared with conventional control (7.9%) [17]. The ADVANCE trial also demonstrated a reduction in microvascular events, mainly driven by a reduction in the progression of albuminuria, but no significant decrease in macrovascular events with intensive glucose control (mean HbA1c of 6.5%) for 5 years, compared with standard control (7.3%) [5]. The Action to Control Cardiovascular Risk in Diabetes (ACCORD) trial revealed that intensive therapy targeting HbA1c < 6.0% for 3.5 years increased mortality and did not significantly reduce major cardiovascular events, compared with standard therapy targeting HbA1c of 7.0–7.9% [6]. Of note, heterogeneity was found among prespecified subgroups, suggesting that patients who had not had a cardiovascular event before randomization may have had fewer cardiovascular events with intensive therapy. The Veterans Affairs Diabetes Trial (VADT) targeting patients with poorly controlled DM found that intensive glucose control (median HbA1c of 6.9%) for 5.6 years had no significant effects on the rates of major cardiovascular events, death, or even microvascular complications, compared with standard therapy (8.4%) [7].

Interestingly, post-trial follow-up for 10 additional years in the UKPDS trial showed the continued reduction in microvascular risk, and emergence of the reduction in myocardial infarction as well as all-cause mortality, although no significant difference in HbA1c levels was present 1 year after the end of the trial [18]. Slow degradation of advanced glycation end products with intensive glycemic control was suggested as one of the plausible mechanisms for this so-called legacy effect. In contrast, observational follow-up for nearly 10 additional years after the VADT ended did not show a mortality benefit with intensive glucose control over the full follow-up period [19]. In addition, intensive glucose control showed a significantly lower risk of major cardiovascular events (HR 0.83, 95% CI 0.70–0.99) only during the prolonged period in which HbA1c curves were separated, disputing the legacy effect [19]. One important difference between the UKPDS and ADVANCE/ACCORD/VADT was that the former was conducted in patients with newly diagnosed DM, whereas the letter included patients who had DM for 8–11.5 years. Therefore, no matter how positively we interpret the above four major trials, modest improvement of macrovascular outcomes might be expected only in patients with newly diagnosed DM, and only after long-term intensive control for at least 10–20 years. In other words, the benefits of intensive glucose control in diabetic patients complicated by cardiovascular disease, who should have a long duration of DM and advanced atherosclerosis, are questionable.

### 4.2. The Impact of Follow-up Glycemic Control after PCI on Clinical Outcomes in Diabetic Patients

A few studies have investigated the impact of intensive glycemic control after PCI on clinical outcomes. Most studies were performed with a small sample size and for a relatively short follow-up duration. Ike et al. compared outcomes in patients with HbA1c < 6.9% at the time of PCI (*n* = 212) with those in patients with HbA1c ≥ 6.9% (*n* = 334) [8]. A 300-day follow-up showed significantly lower incidence of MACE, which was driven by TLR, in HbA1c < 6.9% group than that in HbA1c ≥ 6.9% group. However, HbA1c ≥ 6.9% group had a higher risk profile including higher prevalence of prior coronary artery bypass graft and three-vessel disease, smaller minimal lumen diameter, and higher diameter stenosis. Multivariate analysis did not show any significant impact of HbA1c or the difference between baseline HbA1c and HbA1c measured at a follow-up time point, which was not clearly specified, on clinical outcomes. Kassaian et al. evaluated 703 diabetic patients who underwent PCI in a single center [9]. Based on their mean HbA1c levels calculated from three HbA1c measurements at 0, 1, and 6 months following PCI, patients were categorized into two groups: good glycemic control (HbA1c ≤ 7%) group (*n* = 291) and poor glycemic control (HbA1c > 7%) group (*n* = 412). Multivariate analysis revealed that poor glycemic control was associated with a significantly higher risk of MACE during a 1-year follow-up, which was mainly driven by target vessel revascularization (HR 2.1, 95% CI 1.10–3.95). Hwang et al. reported the association between glycemic control after PCI and clinical outcomes among 980 diabetic patients who underwent PCI in a single center [10]. Based on the HbA1c level measured at 2-year follow-up point, patients were categorized into two groups: HbA1c < 7.0 group (*n* = 489) and HbA1c ≥ 7.0 group (*n* = 491). HbA1c < 7.0 group was associated with a lower risk of major adverse cardiac and cerebrovascular events, mainly driven by repeat revascularization during median follow-up of 5.4-years. However, this study was based on a single center experience, and the study groups were classified according to HbA1c measured at a single time point. Some other studies demonstrated that higher HbA1c measured before or at the time of PCI was associated with poor clinical outcomes [20,21,22,23,24]. However, the impact of intensive glycemic control after PCI was not dealt with in these studies.

To our best knowledge, our study analyzed the largest number of patients (a total of 2576 patients, and 516 matched pairs) on this subject, using a patient-level pooled registry consisting of five multicenter prospective DES registries. Our data reflect the results of contemporary medication and interventional technology. Moreover, this is the first study in which medical records regarding HbA1c were meticulously gathered on a trimonthly basis throughout the follow-up period after discharge and the mean value of HbA1c was used to categorize the patients. By using this approach, patients’ glycemic control levels could be assessed more accurately than ever.

### 4.3. Benefits of New Generation Glucose-Lowering Agents: SGLT2 Inhibitors and GLP-1 Receptor Agonists

Recently, new types of anti-diabetic drugs such as sodium-glucose co-transporter 2 (SGLT2) inhibitors and glucagon-like peptide-1 (GLP-1) agonists were introduced. Treatment with SGLT2 inhibitors significantly decreased the occurrence of composite primary outcomes [25,26,27]. Empagliflozin reduced cardiovascular death by 38% and heart failure hospitalization by 35% [25]. Mechanisms underlying these benefits are still unclear. However, the glucose-lowering effect of empagliflozin seems unlikely to explain them [28]. Because the adjustment of glucose-lowering therapy was encouraged to achieve desired glycemic control in both empagliflozin and control groups, differences in HbA1c between the two groups were minimal. Furthermore, the incidence of MI or stroke, which could be affected by better glycemic control, did not change with empagliflozin treatment. Especially, the curves of clinical outcomes separated in the first months after randomization, which was exceptionally early, given that translation of glycemic control into improved clinical outcomes takes more than several years.

Among GLP-1 agonists, liraglutide and semaglutide demonstrated significant reduction in cardiovascular events [29,30,31]. The underlying mechanism for this benefit also has not been established. Clinical benefit might be derived from reduction in blood pressure, body weight, and low-density lipoprotein cholesterol, rather than better glycemic control [15,32]. Interestingly, the cardiovascular benefits of SGLT2 inhibitors or GLP-1 agonists were more evident in subjects with established atherosclerotic cardiovascular disease or higher risk of that [33], which was in contrast to the lessons from UKPDS/ADVANCE/ACCORD/VADT trials. Since SGLT2 inhibitors or GLP-1 agonists were not available during the period of enrolment, we could not assess the impact of these drugs on clinical outcomes. However, elucidation of the clinical implication of these drugs, which may be related to their pleiotropic effects, is beyond the scope of our study because the aim of this study was to reveal the impact of intensive glycemic control per se after PCI.

### 4.4. Limitations

There are several limitations to our study. First, this study has the intrinsic limitations of a nonrandomized registry-based study such as allocation bias, different distribution of risk factors, and possible influences from unmeasured confounding factors, although we performed Cox regression analysis and propensity score matching to overcome these limitations. Second, because we included patients with ≥2 records of HbA1c during the follow-up period, this may lead to a potential selection bias. Third, the median duration of follow-up was 33.6 months. There is a possibility that a follow-up duration much longer than ours is necessary to find significant effects of intensive glucose control in patients undergoing PCI. However, the impact of glycemic control on macrovascular complications in patients with long durations of DM and advanced vascular disease has been questioned. Fourth, there was no information regarding the duration of DM in our database. However, because the study subjects had established cardiovascular disease, and underwent PCI, we can reasonably speculate that most patients had been diabetic for signification duration. Fifth, we could not get information regarding types of DM. However, since the median age of the study subjects was 66 and 80.3% of patients did not take insulin, we guess that the vast majority had type 2 DM. Moreover, improved glycemic control is considered to result in a larger cardiovascular risk reduction in patients with type 1 DM than that in patients with type 2 DM [18]. Therefore, any potential inclusion of type 1 diabetic patients into the study population would probably lead to an overestimation of the association between intensive glycemic control and better clinical outcomes. This speculation further emphasizes that intensive glycemic control after PCI was not associated with better outcomes. Sixth, although we considered comorbidities, concomitant medications including aspirin and statin, and baseline laboratory results for lipid profile, temporal variation in these data could not be considered in our study. Finally, the cardioprotective effects of new generation glucose-lowering agents, SGLT-2 inhibitors, and GLP-1 receptor agonists could not be assessed in our study. However, as mentioned above, this subject is beyond the scope of our study.

## 5. Conclusions

Strict glycemic control (mean HbA1c 6~7%) during 3-year follow-up period after PCI was not associated with better clinical outcomes than lenient control (mean HbA1c 7~9%) in diabetic patients with pre-existing coronary artery disease after PCI. These data suggest that intensity of glucose control may not be the major factor determining mid-term prognosis after PCI in diabetic patients.

## Figures and Tables

**Table 1:** Baseline characteristics of diabetic patients according to glycemic control based on HbA1c 7.0% during follow-up period after PCI.

	Overall Population				PS Matched Population			
	Mean HbA1c < 7.0% (N = 1087)	Mean HbA1c ≥ 7.0% (N = 1489)	*p* Value	ASD	Mean HbA1c < 7.0% (N = 516)	Mean HbA1c ≥ 7.0% (N = 516)	*p* Value	ASD
**Demographics**								
Age	67 (59–73)	66 (58–72)	0.190	0.050	67 (58–73)	66 (59–72)	0.733	0.059
Male	734 (67.5)	957 (64.3)	0.093	0.033	343 (66.5)	350 (67.8)	0.691	0.008
BMI, kg/m^2^	24.7 ± 3.1	24.9 ± 3.2	0.296	0.042	24.9 ± 3.1	25.0 ± 3.0	0.696	0.022
Follow-up duration, days	990 (630–1113)	990 (630–1114)	0.317					
**Comorbidities**								
Hypertension	808 (74.3)	1069 (71.8)	0.164	0.025	376 (72.9)	381 (73.8)	0.778	0.025
Dyslipidemia	430 (39.6)	604 (40.6)	0.625	0.010	204 (39.5)	217 (42.1)	0.447	0.019
Current smoking	264 (24.3)	383 (25.7)	0.434	0.014	123 (23.8)	123 (23.8)	0.999	0.008
Heart failure or LVEF < 40%	90 (8.3)	158 (10.6)	0.050	0.023	54 (10.5)	51 (9.9)	0.837	0.004
MI or revascularization	236 (21.6)	303 (20.3)	0.405	0.014	107 (20.7)	115 (22.3)	0.596	0.008
Chronic kidney disease	82 (7.6)	119 (8.0)	0.710	0.004	38 (7.4)	40 (7.8)	0.906	0.004
Peripheral vascular disease	31 (2.9)	43 (2.9)	0.999	<0.001	15 (2.9)	14 (2.7)	0.999	0.002
Previous stroke	108 (9.9)	155 (10.4)	0.742	0.005	57 (11.0)	48 (9.3)	0.410	0.006
Initial presentation with acute MI	294 (27.1)	423 (28.5)	0.449	0.014	138 (26.7)	142 (27.5)	0.834	0.029
**Lesion and Procedure Characteristics**								
Multivessel disease	396 (36.5)	477 (32.0)	0.021	0.044	176 (34.1)	185 (35.9)	0.602	0.008
Left main disease	88 (8.1)	110 (7.4)	0.502	0.007	35 (6.8)	36 (7.0)	0.999	0.002
Type B2/C lesions	913 (84.1)	1217 (81.7)	0.126	0.024	426 (82.6)	429 (83.1)	0.869	0.016
Calcification	87 (8.0)	119 (8.0)	0.999	<0.001	38 (7.4)	44 (8.5)	0.565	0.017
2nd generation DES	1010 (92.9)	1359 (91.1)	0.105	0.018	467 (90.5)	475 (92.1)	0.440	0.004
Total stent number	1.8 ± 1.1	1.7 ± 1.0	0.088	0.066	1.7 ± 1.0	1.8 ± 1.0	0.395	0.015
Stent diameter, mm	3.0 ± 0.4	3.0 ± 0.4	0.694	0.016	3.0 ± 0.4	3.0 ± 0.4	0.632	0.048
Total stent length, mm	44.8 ± 29.6	42.1 ± 27.8	0.022	0.089	42.8 ± 27.9	44.5 ± 30.2	0.326	0.017
**Medications**								
Insulin	198 (18.2)	309 (20.8)	0.120	0.025	104 (20.2)	100 (19.4)	0.815	0.006
Sulfonylurea	424 (39.0)	706 (47.4)	<0.001	0.084	222 (43.0)	236 (45.7)	0.415	0.019
Glinide	13 (1.2)	26 (2.4)	0.036	0.012	10 (1.9)	10 (1.9)	0.999	<0.001
Metformin	571 (52.6)	797 (53.5)	0.632	<0.001	271 (52.5)	272 (52.7)	0.999	<0.001
DPP4i	201 (18.5)	259 (17.4)	0.498	0.011	92 (17.8)	107 (20.7)	0.269	0.010
Thiazolidinedione	31 (2.9)	42 (2.8)	0.999	<0.011	17 (3.3)	18 (3.5)	0.999	0.006
a-glucosidase inhibitor	73 (6.7)	126 (8.5)	0.118	0.017	41 (7.9)	34 (6.6)	0.472	0.017
Aspirin	1082 (99.5)	1482 (99.5)	0.999	<0.001	512 (99.2)	514 (99.6)	0.682	0.006
Clopidogrel	1066 (98.1)	1459 (98.0)	0.955	0.001	503 (97.5)	504 (97.7)	0.999	<0.001
Beta-blockers	660 (60.7)	949 (63.7)	0.128	0.030	324 (62.8)	323 (62.6)	0.999	0.004
ACE inhibitors	296 (27.2)	464 (31.2)	0.034	0.039	152 (29.5)	147 (28.5)	0.784	0.014
ARBs	415 (38.2)	573 (38.5)	0.908	0.003	183 (35.5)	199 (38.6)	0.334	0.006
Statin	948 (87.2)	1289 (86.6)	0.680	0.006	452 (87.6)	447 (86.6)	0.710	0.016
**Laboratory Results**								
Baseline HbA1c, %	6.5 ± 0.8	8.1 ± 1.5	<0.001	2.074	7.0 ± 0.8	7.0 ± 1.0	0.397	0.061
Numbers of HbA1c records	5.7 ± 3.3	6.2 ± 3.5	<0.001	0.161	6.2 ± 3.3	6.3 ± 3.4	0.624	0.024
Total cholesterol, mg/dL	158.5 ± 40.1	163.7 ± 43.4	0.002	0.128	163.1 ± 41.3	161.4 ± 40.5	0.505	0.028
Triglyceride, mg/dL	122.3 ± 95.5	127.1 ± 112.4	0.240	0.051	123.3 ± 98.2	122.9 ± 100.6	0.951	0.038
HDL, mg/dL	35.0 ± 18.2	34.8 ± 18.4	0.803	0.010	34.2 ± 19.0	35.2 ± 18.4	0.389	0.002
LDL, mg/dL	77.0 ± 47.8	78.8 ± 50.3	0.359	0.037	77.9 ± 51.3	77.5 ± 50.6	0.910	0.028
Creatinine clearance, ml/min	67.7 ± 28.7	66.9 ± 29.2	0.468	0.029	67.2 ± 28.2	67.1 ± 28.8	0.954	0.013

Values given as mean ± standard deviation, median (interquartile range, 25th and 75th percentile), or number (percentage), unless otherwise indicated; Abbreviations: ACE, angiotensin-converting enzyme; ARBs, angiotensin II receptor blockers; ASD, absolute standardized difference; BMI, body mass index; DES, drug eluting stent; DPP4i, dipeptidyl peptidase-4 inhibitor; HDL, high-density lipoprotein; LDL, low-density lipoprotein; LVEF, Left ventricular ejection fraction; MI, Myocardial infarction; PS, propensity score.

**Table 2:** Comparison of clinical outcomes after PCI depending on follow-up glucose control based on HbA1c 7.0%.

Outcome	Overall Population				PS Matched Population			
	Mean HbA1c < 7.0% (N = 1087)	Mean HbA1c ≥ 7.0% (N = 1489)	aHR (95% CI)	*p* Value	Mean HbA1c < 7.0% (N = 516)	Mean HbA1c ≥ 7.0% (N = 516)	aHR (95% CI)	*p* Value
	N (%)	N (%)			N (%)	N (%)		
MACE	138 (12.7)	197 (13.2)	1.06 (0.82–1.37)	0.672	73 (14.1)	63 (12.2)	1.17 (0.84–1.65)	0.351
All-cause death	56 (5.2)	79 (5.3)	1.14 (0.75–1.72)	0.553	33 (6.4)	23 (4.5)	1.44 (0.85–2.46)	0.177
Cardiac death	34 (3.1)	52 (3.5)	1.06 (0.63–1.81)	0.820	21 (4.1)	12 (2.3)	1.76 (0.87–3.36)	0.119
Non-fatal MI	6 (0.6)	22 (1.5)	0.41 (0.15–1.16)	0.093	3 (0.6)	6 (1.2)	0.50 (0.13–2.01)	0.330
Cardiac death + non-fatal MI	40 (3.7)	73 (4.9)	0.90 (0.57–1.42)	0.639	24 (4.7)	18 (3.5)	1.34 (0.72–2.46)	0.354
Stroke	19 (1.7)	16 (1.1)	1.5 (0.68–3.59)	0.295	8 (1.6)	4 (0.8)	2.00 (0.60–6.65)	0.257
Any revascularization	104 (9.8)	145 (9.7)	1.05 (0.78–1.42)	0.748	53 (10.3)	52 (10.1)	1.03 (0.71–1.52)	0.866
Target lesion revascularization	49 (4.5)	63 (4.2)	1.12 (0.71–1.78)	0.615	26 (5.0)	23 (4.5)	1.15 (0.65–2.01)	0.634

Abbreviations: CI, confidence interval; aHR, adjusted hazard ratio; MACE, major adverse cardiovascular event; MI, myocardial infarction; PS, propensity score.

**Table 3:** The impact of stable glycemic control during follow-up on clinical outcome after PCI.

Outcome	Sustained HbA1c < 7.0% (N = 716)	Sustained HbA1c ≥ 7.0% (N = 532)		aHR (95% CI)	*p* Value
	N (%)	N (%)			
MACE	62 (8.7)	91 (17.1)	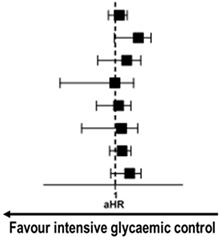	1.15 (0.71–1.89)	0.566
All-cause Death	22 (3.1)	27 (5.1)		2.70 (0.95–6.70)	0.057
Cardiac Death	13 (1.8)	21 (3.9)		1.26 (0.40–3.97)	0.698
Non-fatal MI	1 (0.1)	12 (2.3)		0.28 (0.03–3.09)	0.301
Cardiac Death + Non-fatal MI	14 (2.0)	33 (6.2)		0.90 (0.35–2.27)	0.816
Stroke	9 (1.6)	10 (1.9)		0.77 (0.17–3.45)	0.730
Any revascularization	51 (7.1)	69 (13.0)		1.34 (0.76–2.34)	0.308
Target lesion revascularization	28 (3.9)	33 (6.2)		1.85 (0.83–4.12)	0.135
				

Abbreviations: CI, confidence interval; aHR, adjusted hazard ratio; MACE, major adverse cardiovascular event; MI, myocardial infarction.

**Table 4:** The impact of more intensive glycemic control during follow-up on clinical outcome after PCI.

Outcome	Mean HbA1c ≤ 6.5%	Mean HbA1c ≥ 8.0%		aHR (95% CI)	*p* Value
	N (%)	N (%)			
MACE	67 (12.2)	79 (12.4)	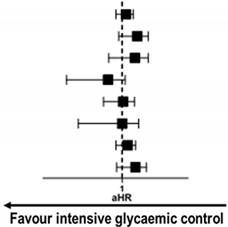	1.15 (0.71–1.86)	0.583
All-cause Death	28 (5.1)	29 (4.6)		1.72 (0.75–3.90)	0.198
Cardiac Death	16 (2.9)	18 (2.8)		1.42 (0.47–4.24)	0.531
Non-fatal MI	2 (0.4)	12 (1.9)		0.19 (0.03–1.35)	0.097
Cardiac Death + Non-fatal MI	18 (3.3)	30 (4.7)		0.81 (0.34–1.93)	0.638
Stroke	7 (1.3)	8 (1.3)		0.62 (0.11–3.51)	0.592
Any revascularization	53 (9.7)	60 (9.4)		1.25 (0.72–2.18)	0.428
Target lesion revascularization	28 (5.1)	27 (4.3)		1.84 (0.80–4.27)	0.154
				

Abbreviations: CI, confidence interval; aHR, adjusted hazard ratio; MACE, major adverse cardiovascular event; MI, myocardial infarction.

## Supplementary Materials

The following are available online at https://www.mdpi.com/2077-0383/9/8/2464/s1, Table S1: Baseline characteristics of patients according to glycemic control status with sustained HbA1c < 7.0% or ≥7.0%, Table S2: Baseline characteristics of patients according to glycemic control status with mean HbA1c ≤ 6.5% or ≥8.0%, Figure S1: Absolute standardized differences of covariates before and after propensity score matching, Figure S2: Temporal changes in HbA1c according to glycemic control status with sustained HbA1c < 7.0% or ≥7.0% (2A) and with mean HbA1c ≤ 6.5% or ≥8.0% (2B), Figure S3: Kaplan–Meier curve for clinical outcomes according to glycemic control status with sustained HbA1c < 7.0% or ≥7.0% (3A) and with mean HbA1c ≤ 6.5% or ≥8.0% (3B) in the overall population.
