# Supplementary material for: Impact of Intensive Glucose Control in Patients with Diabetes Mellitus Undergoing Percutaneous Coronary Intervention: 3-Year Clinical Outcomes
Source: J Clin Med. 2020 Aug 1;9(8):2464. doi: 10.3390/jcm9082464 (PMC7465631; doi:10.3390/jcm9082464)
Supplement: Supplementary file 1 [file jcm-09-02464-s001.pdf]

SUPPLEMENTAL MATERIAL

Impact of Intensive Glucose Control in Patients with Diabetes Mellitus Undergoing Percutaneous Coronary Intervention

Table S1. Baseline characteristics of patients according to glycemic control status with sustained HbA1c<7.0% or ≥7.0%

Table S2. Baseline characteristics of patients according to glycemic control status with mean HbA1c≤6.5% or ≥8.0%

Figure S1. Absolute standardized differences of covariates before and after propensity score matching

ACE = angiotensin-converting enzyme; ARBs = angiotensin II receptor blockers; BMI = body mass index; DPP4 = dipeptidyl peptidase-4; HDL = high-density lipoprotein; LDL = low-density lipoprotein; LVEF = Left ventricular ejection fraction; MI = Myocardial infarction.

Figure S2. Temporal changes in HbA1c according to glycemic control status with sustained HbA1c<7.0% or ≥7.0% (2A) and with mean HbA1c≤6.5% or ≥8.0% (2B)

Figure S3. Kaplan-Meier curve for clinical outcomes according to glycemic control status with sustained HbA1c<7.0% or ≥7.0% (3A) and with mean HbA1c≤6.5% or ≥8.0% (3B) in the overall population

MACE = major adverse cardiovascular event.

Table S1. Baseline characteristics of patients according to glycemic control status with sustained HbA1c<7.0% or ≥7.0%

	Sustained HbA1c<7.0% (N=532)	Sustained HbA1c≥7.0% (N=716)	p value
**Demographics**			
Age	67 (59-72)	66 (59-72)	0.160
Male	359 (67.5)	451 (63.0)	0.113
BMI, kg/m^2	24.7±3.2	24.8±3.3	0.611
**Follow-up duration, days**	900 (450-1,080)	990 (540-1,101)	0.021
**Comorbidities**			
Hypertension	413 (77.6)	507 (70.8)	0.008
Dyslipidemia	212 (39.8)	275 (38.4)	0.647
Current smoking	113 (21.2)	184 (25.7)	0.078
Heart failure or LVEF < 40%	42 (7.9)	80 (11.2)	0.067
MI or revascularization	117 (22.0)	135 (18.9)	0.196
Chronic kidney disease	36 (6.8)	55 (7.7)	0.614
Peripheral vascular disease	15 (2.8)	13 (1.8)	0.322
Previous stroke	50 (9.4)	72 (10.1)	0.772
**Initial presentation with acute MI**	128 (24.1)	199 (27.8)	0.156
**Lesion and Procedure characteristics**			
Multivessel disease	191 (35.9)	235 (32.8)	0.282
Left main disease	47 (8.8)	58 (8.1)	0.720

Type B2/C lesions	446 (83.8)	585 (81.7)	0.365
Calcification	38 (7.1)	48 (6.7)	0.849
2nd generation DES	496 (93.2)	643 (89.8)	0.043
Total stent number	1.8±1.0	1.7±1.0	0.495
Stent diameter, mm	3.0±0.4	3.0±0.4	0.887
Total stent length, mm	43.9±27.8	42.0±26.8	0.228
**Medications**			
Insulin	93 (17.5)	155 (21.6)	0.080
Sulfonylurea	194 (36.5)	334 (46.6)	<0.001
Glinide	5 (0.9)	16 (2.2)	0.124
Metformin	272 (51.1)	385 (53.8)	0.386
DPP4i	88 (16.5)	116 (16.2)	0.934
Thiazolidinedione	13 (2.4)	18 (2.5)	0.999
a-glucosidase inhibitor	41 (7.7)	62 (8.7)	0.617
Aspirin	529 (99.4)	713 (99.6)	0.999
Clopidogrel	523 (98.4)	705 (98.5)	0.999
Beta-blockers	314 (59.0)	471 (65.8)	0.017
ACE inhibitors	141 (26.5)	248 (34.6)	0.003
ARBs	218 (41.0)	264 (36.9)	0.157
Statin	456 (85.7)	620 (86.6)	0.717

**Laboratory Results**			
Baseline HbA1c, %	6.2±0.4	8.6±1.3	<0.001
Total cholesterol, mg/dL	159.1±38.7	163.6±44.1	0.054
Triglyceride, mg/dL	119.6±89.1	135.5±118.3	0.007
HDL, mg/dL	35.2±18.4	35.7±17.8	0.649
LDL, mg/dL	76.5±47.3	79.6±48.7	0.245
Creatinine clearance, ml/min	66.6±26.5	66.5±29.1	0.918

Values given as mean ± standard deviation, median (interquartile range, 25th and 75th percentile), or number (percentage), unless otherwise indicated.
Abbreviations: ACE, angiotensin-converting enzyme; ARBs, angiotensin II receptor blockers; BMI, Body mass index; DES, Drug eluting stent; DPP4i, dipeptidyl peptidase-4 inhibitor; HDL, high-density lipoprotein; LDL, low-density lipoprotein; LVEF, Left ventricular ejection fraction; MI, Myocardial infarction.

Table S2. Baseline characteristics of patients according to glycemic control status with mean HbA1c≤6.5% or ≥8.0%

	Mean HbA1c≤6.5% (N=548)	Mean HbA1c≥8.0% (N=635)	p value
**Demographics**			
Age	67 (59-72)	65 (58-72)	0.133
Male	363 (66.2)	386 (60.8)	0.060
BMI, kg/m^2	24.7±3.1	25.0±3.4	0.100
**Follow-up duration, days**	911 (540-1,094)	990 (630-1,106)	0.048
**Comorbidities**			
Hypertension	420 (76.6)	451 (71.0)	0.034
Dyslipidemia	218 (39.8)	247 (38.9)	0.802
Current smoking	123 (22.4)	172 (27.1)	0.076
Heart failure or LVEF < 40%	43 (7.8)	64 (10.1)	0.218
MI or revascularization	114 (20.8)	125 (19.7)	0.686
Chronic kidney disease	41 (7.5)	47 (7.4)	0.999
Peripheral vascular disease	19 (3.5)	14 (2.2)	0.255
Previous stroke	57 (10.4)	66 (10.4)	0.999
**Initial presentation with acute MI**	138 (25.2)	176 (27.7)	0.358
**Lesion and Procedure characteristics**			
Multivessel disease	195 (35.6)	209 (32.9)	0.366
Left main disease	47 (8.6)	57 (9.0)	0.889

Type B2/C lesions	468 (85.4)	513 (80.8)	0.043
Calcification	44 (8.0)	46 (7.2)	0.691
2nd generation DES	513 (93.6)	569 (89.6)	0.019
Total stent number	1.8±1.0	1.7±1.0	0.427
Stent diameter, mm	3.0±0.4	3.0±0.4	0.779
Total stent length, mm	44.2±27.9	41.6±27.0	0.097
**Medications**			
Insulin	93 (17.0)	156 (24.6)	0.002
Sulfonylurea	193 (35.2)	313 (49.3)	<0.001
Glinide	6 (1.1)	12 (1.9)	0.3881
Metformin	289 (52.7)	345 (54.3)	0.624
DPP4i	104 (19.0)	90 (14.2)	0.032
Thiazolidinedione	11 (2.0)	18 (2.8)	0.466
a-glucosidase inhibitor	32 (5.8)	57 (9.0)	0.054
Aspirin	545 (99.5)	635 (100.0)	0.198
Clopidogrel	539 (98.4)	626 (98.6)	0.939
Beta-blockers	318 (58.0)	412 (64.9)	0.018
ACE inhibitors	146 (26.6)	222 (35.0)	0.003
ARBs	224 (40.9)	232 (36.5)	0.142
Statin	471 (85.9)	557 (87.7)	0.417

**Laboratory Results**			
Baseline HbA1c, %	6.2±0.6	8.9±1.6	<0.001
Total cholesterol, mg/dL	159.3±39.3	163.8±44.1	0.058
Triglyceride, mg/dL	118.0±90.6	136.0±115.6	0.011
HDL, mg/dL	35.1±19.1	35.3±18.0	0.840
LDL, mg/dL	95.4±47.8	79.8±49.5	0.128
Creatinine clearance, ml/min	66.0±27.3	66.8±30.4	0.602

Values given as mean ± standard deviation, median (interquartile range, 25th and 75th percentile), or number (percentage), unless otherwise indicated.
Abbreviations: ACE, angiotensin-converting enzyme; ARBs, angiotensin II receptor blockers; BMI, Body mass index; DES, Drug eluting stent; DPP4i, dipeptidyl peptidase-4 inhibitor; HDL, high-density lipoprotein; LDL, low-density lipoprotein; LVEF, Left ventricular ejection fraction; MI, Myocardial infarction.

Figure S1

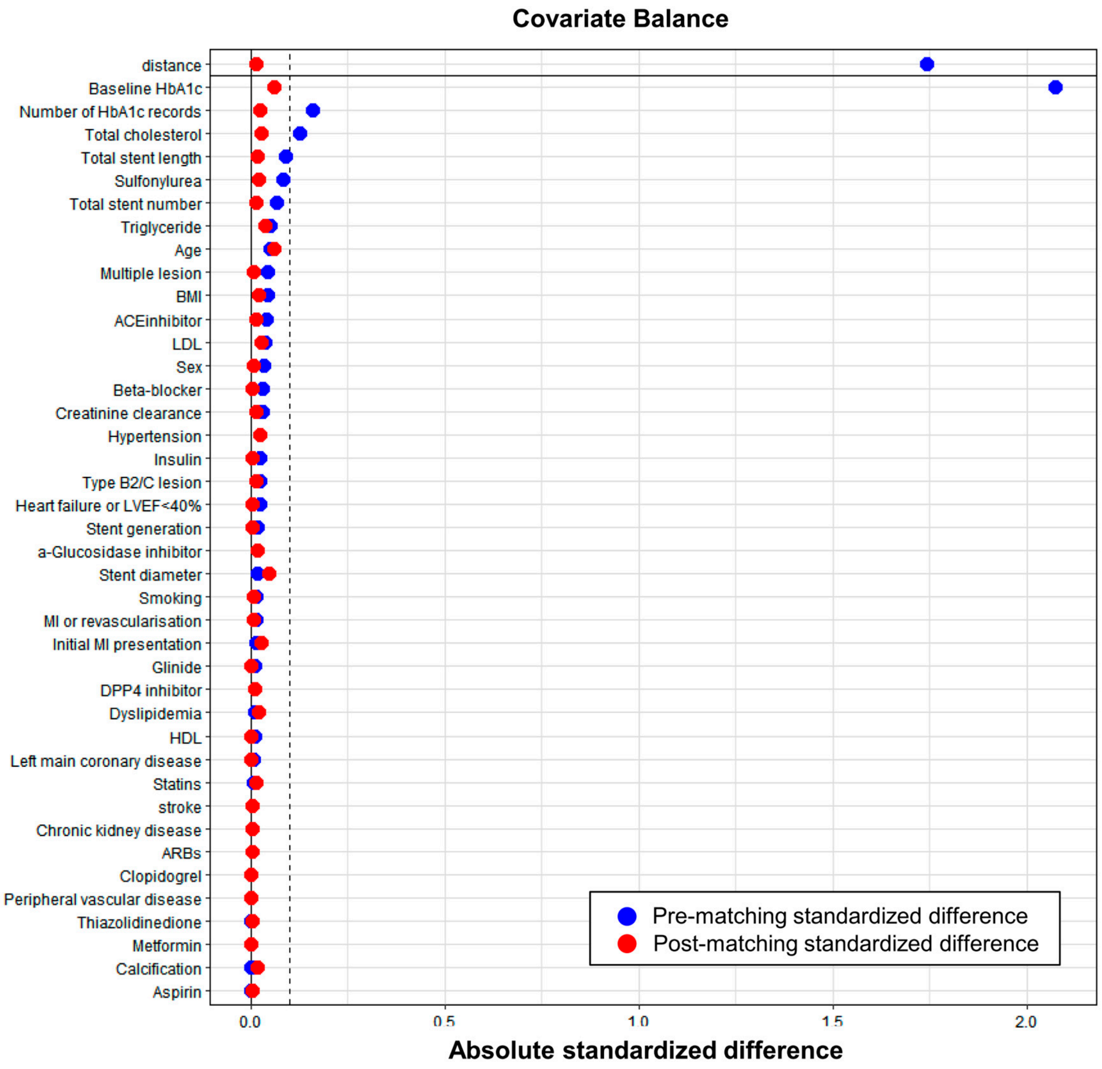

Covariate Balance
distance
Baseline HbA1c
Number of HbA1c records
Total cholesterol
Total stent length
Sulfonylurea
Total stent number
Triglyceride
Age
Multiple lesion
BMI
ACEinhibitor
LDL
Sex
Beta-blocker
Creatinine clearance
Hypertension
Insulin
Type B2/C lesion
Heart failure or LVEF<40%
Stent generation
a-Glucosidase inhibitor
Stent diameter
Smoking
MI or revascularisation
Initial MI presentation
Glinide
DPP4 inhibitor
Dyslipidemia
HDL
Left main coronary disease
Statins
stroke
Chronic kidney disease
ARBs
Clopidogrel
Peripheral vascular disease
Thiazolidinedione
Metformin
Calcification
Aspirin
0.0
0.5
1.0
1.5
2.0
Absolute standardized difference
Pre-matching standardized difference
Post-matching standardized difference

Figure S2

2A.

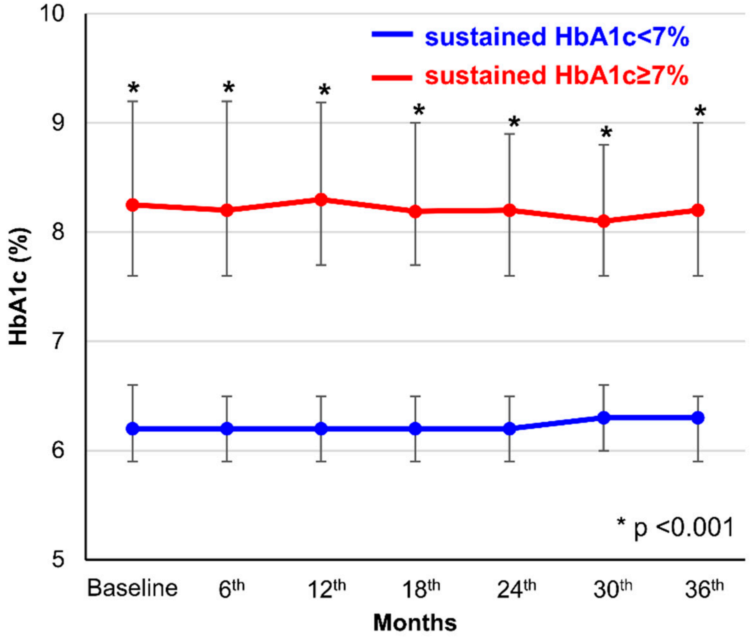

Numbers of patients with HbA1c record

Follow-up Months	Baseline	6th	12th	18th	24th	30th	36th
sustained HbA1c<7%	532	190	167	147	141	125	146
sustained HbA1c≥7%	716	302	292	269	248	244	241

2B.

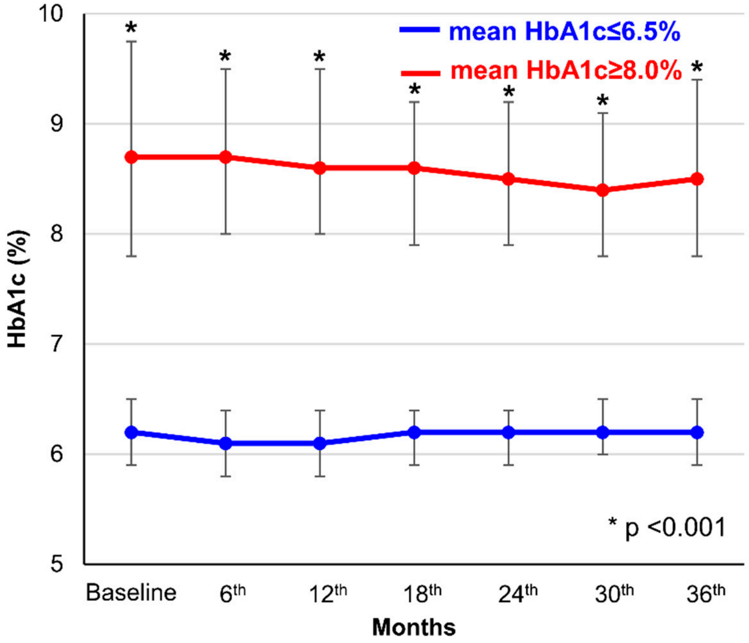

Numbers of patients with HbA1c record

Follow-up Months	Baseline	6th	12th	18th	24th	30th	36th
mean HbA1c≤6.5%	548	236	198	195	177	170	185
mean HbA1c≥8.0%	635	289	288	265	244	237	230

Figure S3

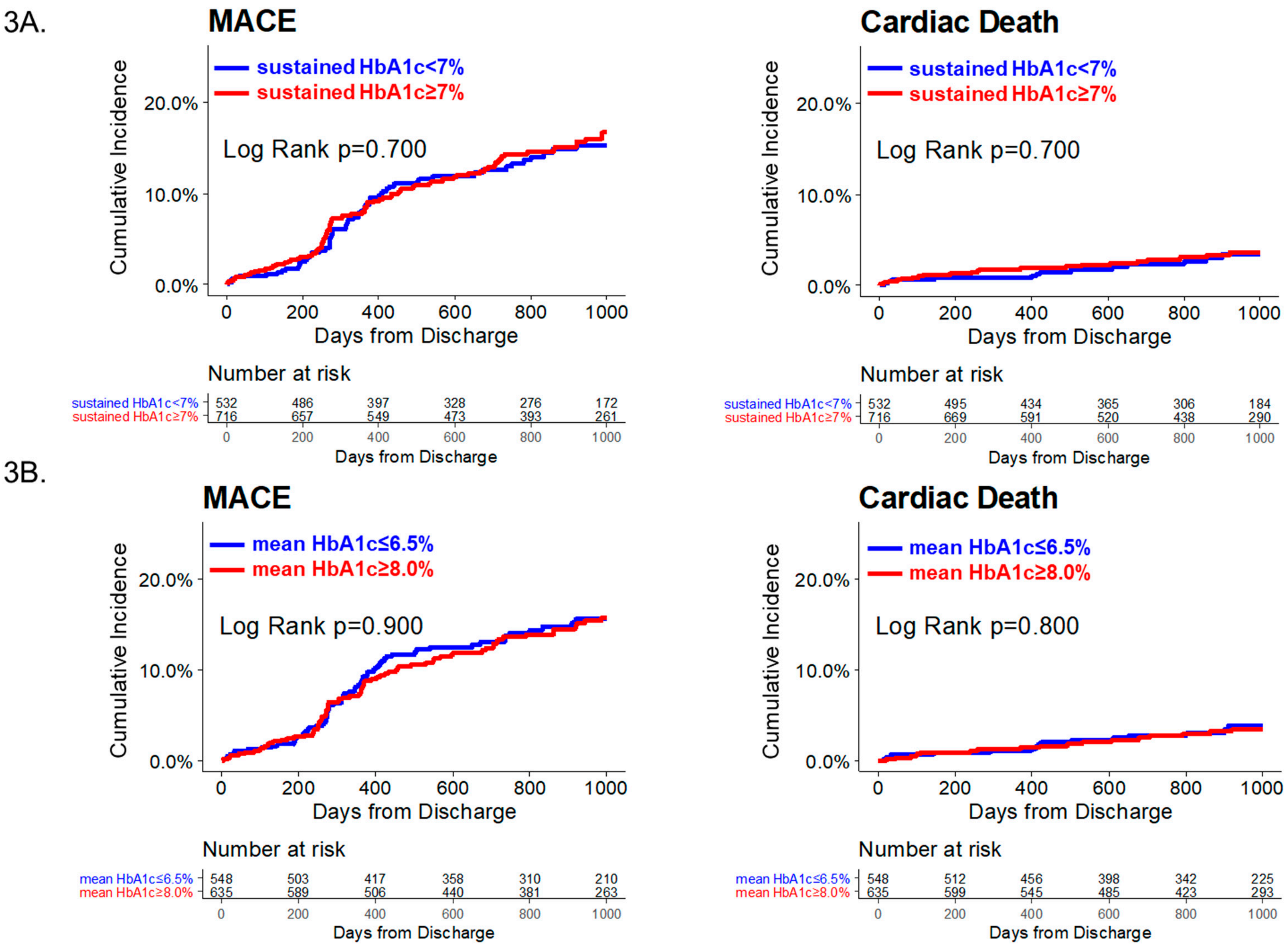
3A.
MACE
sustained HbA1c<7%
sustained HbA1c≥7%
Log Rank p=0.700
Cumulative Incidence
0.0%
10.0%
20.0%
0
200
400
600
800
1000
Days from Discharge
Number at risk
sustained HbA1c<7% 532 486 397 328 276 172
sustained HbA1c≥7% 716 657 549 473 393 261
Days from Discharge
Cardiac Death
sustained HbA1c<7%
sustained HbA1c≥7%
Log Rank p=0.700
Cumulative Incidence
Days from Discharge
Number at risk
sustained HbA1c<7% 532 495 434 365 306 184
sustained HbA1c≥7% 716 669 591 520 438 290
Days from Discharge
3B.
MACE
mean HbA1c≤6.5%
mean HbA1c≥8.0%
Log Rank p=0.900
Cumulative Incidence
Days from Discharge
Number at risk
mean HbA1c≤6.5% 548 503 417 358 310 210
mean HbA1c≥8.0% 635 589 506 440 381 263
Days from Discharge
Cardiac Death
mean HbA1c≤6.5%
mean HbA1c≥8.0%
Log Rank p=0.800
Cumulative Incidence
Days from Discharge
Number at risk
mean HbA1c≤6.5% 548 512 456 398 342 225
mean HbA1c≥8.0% 635 599 545 485 423 293
Days from Discharge
